# Carbon Ion Fluxes at Mars: First Results of Tailward Flows From MAVEN‐STATIC

**DOI:** 10.1029/2021JA029635

**Published:** 2021-12-30

**Authors:** N. B. Pickett, J. P. McFadden, C. M. Fowler, K. G. Hanley, M. Benna

**Affiliations:** ^1^ Space Sciences Laboratory Berkeley CA USA; ^2^ Goddard Space Flight Center Greenbelt MD USA

**Keywords:** carbon escape, Mars ionosphere, MAVEN‐STATIC, carbon ion escape, STATIC instrument calibration

## Abstract

Characterizing C^+^ ions in the Martian ionosphere is important for understanding the history of the Martian atmosphere and surface due to its place in understanding carbon escape. Measuring minor ions, like C^+^, which are close in mass to major atmospheric ions, in this case O^+^, is difficult, requiring fitting algorithms and accurate background subtraction. Accurate measurement of these species is essential for understanding chemistry and transport in the ionosphere. In this paper, we use data from the Mars Atmospheric and Volatile EvolutioN SupraThermal And Thermal Ion Composition (MAVEN‐STATIC) sensor to report the first C^+^ fluxes measured in the Martian magnetotail. We will describe a multistep method of background subtraction as well as fitting routines that are used to extract C^+^ fluxes from a 40‐orbit subset of STATIC data. Our results show tailward fluxes in both optical shadow and the adjacent sunlit magnetotail at high altitudes (>3,000 km) and Mars‐ward at low altitudes (<2,000 km) in shadow. These local flux values are similar to estimates of neutral carbon fluxes from photochemical escape. However, total carbon loss comparisons will require a more comprehensive study of integrated C^+^ loss over a larger data set from the Martian magnetotail.

## Key Points

- C^+^ fluxes were detected 27% of the time above a 2.2 sigma noise threshold during 40 orbits throughout the Mars magnetotail
- Typical C^+^ fluxes (10^4^ − 10^5^/cm^2^ s) are 1–2 orders of magnitude lower than either O^+^ or O_2_^+^ fluxes
- Fluxes are tailward at altitudes >3,000 km and Mars‐ward at <2,000 km

## 1 Introduction

Since the discovery of geological evidence of glaciation and other water phenomena on the surface of Noachian Mars (Baker, 2001), research has indicated that the Martian atmosphere has drastically changed over time, as only small transient flows of perchlorate brine are known to exist on the modern Martian surface due to low pressure (Martin‐Torres et al., 2015). There is abundant evidence that this climate evolution has occurred over the course of Mars' history, is still ongoing today, and has been driven at least in part by loss of the atmosphere to space (Chassefière & Leblanc, 2004; Jakosky et al., 2015; Lundin et al., 2004). Understanding the loss of Mars' atmosphere to space is thus crucial to understanding the long‐term habitability and evolution of the climate as a whole.

Light atmospheric species (such as hydrogen) can be lost to space via thermal escape mechanisms due to their low mass. For the heavier species like C^+^ and CO_2_^+^, however, energies of 1.5 and 6 eV, respectively, are required to overcome Mars' gravitational potential. This means that some additional acceleration mechanism is required for heavy species to escape (Chassefière & Leblanc, 2004). This escape energy, high enough to attain escape velocities (Brain et al., 2015; Lillis et al., 2017; Lundin et al., 2004), can be obtained through photochemical reactions (Fox & Hać, 2009), where exothermic reactions produce “hot” neutral atoms that are able to escape, or when charged particles experience electromagnetic forces.

To our knowledge, little work has been published on the loss of C^+^ ions from the Martian ionosphere. Their importance in the production of neutral carbon, via photochemical reactions and charge exchange, has been noted by, e.g., Fox and Bakalian (2001) and Lo et al. (2020). C^+^ ions are primarily produced via the photodissociative ionization of CO_2_ (Lo et al., 2020), and subsequent photoionization of C; however, C^+^ ions were not the focus of that work and other production channels may exist.

Once heavy ions are produced in the Martian ionosphere, they can be subject to a variety of electromagnetic forces that can energize them to escape energy and overcome the gravitational potential (1.5 eV for C^+^ at Mars). Processes include pickup by the solar wind (Dong et al., 2015a; Lundin et al., 2008), wave heating (Ergun et al., 2006; Nilsson et al., 2012), acceleration by ambipolar electric fields (Collinson et al., 2015; Ergun et al., 2016), and bulk escape related to the crustal magnetic field (Brain et al., 2010). Most studies of escaping ions at Mars have focused on the escape of the most abundant ions, namely O^+^ and O_2_^+^ (Curry et al., 2015; Daerden et al., 2019; Dong et al., 2015; Ergun et al., 2016; Girazian et al., 2019; Kar et al., 1996; Kim et al., 1998; Ramstad et al., 2015), while few studies exist related to the escape of minor atmospheric ion species.

This study focuses on quantifying the escape of the minor singularly charged ion species carbon (C^+^), presenting the first calculations of C^+^ escape fluxes at Mars, derived from data collected by the MAVEN‐STATIC instrument. Mars' atmosphere predominantly comprises neutral CO_2_ and understanding the escape of carbon to space is thus necessary in order to fully understand the evolution of Mars' climate. It is thought that loss to space is an important carbon sink in understanding the history of Mars (Hu et al., 2015; Jakosky et al., 2018), and modeling work has obtained estimates for the photochemical escape rates of neutral carbon (Fox & Bakalian, 2001; Lo et al., 2021). However, no known studies have investigated the escape of carbon ions from Mars, in part because no orbiter prior to MAVEN has had the capability to reliably detect C^+^.

It has been estimated that Mars has lost at least 0.8 bar of CO_2_ from known atmospheric loss mechanisms (Jakosky et al., 2018). This estimate stems from one of several novel Martian atmosphere loss models that use recent MAVEN data to clarify Mars' carbon budget (Daerden et al., 2019; Jakosky et al., 2018; Lo et al., 2020). Earlier models such as Fox and Bakalian (2001) account for C^+^ being made by photodissociative ionization of CO. Later models such as in Lo et al. (2020) take into account thousands of unique photochemical reactions between neutral and ion species to find what reaction paths lead to the significant carbon escape. These models show charge‐exchanging reactions with heavy ions in the ionosphere are responsible for producing more escaping neutral carbon than previously known, particularly reactions with C^+^ (Lo et al., 2020). This ion species, while part of the discussion of neutral photochemical escape, also itself escapes via electromagnetic forces that send it down Mars' magnetotail.

In this study, we will use STATIC data to determine the outflow of C^+^ ions on Mars. Our analysis is built on methods used to perform background subtraction on STATIC data. While this method is very specific to C^+^ and STATIC, the base procedures could inform future methods of extracting other heavy ions. Section 2 will break down the instrument and data used. Section 3 will go through the method used to isolate C^+^. Section 4 will look at results, which will be analyzed and discussed in Section 5. Section 6 will summarize our results and look forward to the future application of this method.

## 2 Instrument and Data

The instrument used for this study, STATIC, is aboard the MAVEN satellite that has been in orbit on Mars collecting data since September 2014 (Jakosky et al., 2015). So far the satellite has completed over 13,000 orbits providing ion measurements from three instruments: STATIC (McFadden et al., 2015), the Neutral Gas and Ion Mass Spectrometer (NGIMS) (Mahaffy et al., 2015), and the Solar Wind Ion Analyzer (SWIA) (Halekas et al., 2015). SWIA provides high energy and angular resolution of the solar wind flow, but cannot resolve ion mass. NGIMS is able to resolve C^+^ ions in the lower ionosphere (<500 km) when ion densities are relatively large, but lacks the sensitivity to observe the more diffuse ion populations. STATIC has the needed mass resolution and dynamic range to observe C^+^ ions throughout MAVEN's orbit, from the cold, dense ionosphere through to energetic fluxes at higher altitudes within the magnetosphere.

### 2.1 STATIC

STATIC's purpose is to measure the thermal and higher‐energy ion populations with regards to their distribution and composition in the Martian ionosphere. The sensor has a 360° × 90° field of view (FOV) and measures ion energy (0.1 eV–30 keV) and mass (0.5–100 amu, mass resolution *M*/d*M* = 4) in a 64 energy × 64 mass × 256 solid angle array every 4 s. These data are summed over various array dimensions to reduce data volume and generate six primary data products that are transmitted back to Earth (McFadden et al., 2015). STATIC functions in both ion‐dense and tenuous regions through the use of attenuators that permit and restrict the incoming ions to prevent saturation, and operates in several different “modes” which have different energy ranges and time resolutions.

The time‐of‐flight (TOF) analyzer aboard STATIC uses two carbon foils to release electrons that determine the time an ion takes to cross a 2‐cm gap. The TOF analyzer accelerates ions to 15 keV before passing them through this pair of carbon foils. Electrons released by the foils serve as a start and stop signal as the ions go through each foil. Since the velocity analyzer measures mass/charge, ions with the same mass/charge like He^++^ and H_2_^+^ ions have the same TOF and individual events cannot be distinguished without more detailed analysis.

### 2.2 Data

STATIC's ability to measure the distribution of ion masses, energies, and velocities every 4 s enables the isolation of C^+^ ions from the data. Due to downlink limitations, the high dimensionality of the data necessitates its division into a number of different data products distinguished by their binning resolution in mass, energy, and solid angle of entry (McFadden et al., 2015). The primary data product used in this work is labeled as “c6” and is used since it has the highest mass resolution at the expense of having no angular resolution. Additionally, it is available at a 4‐s cadence for the entire mission. As will be discussed later, this high resolution is needed in order for the background removal programs to successfully fit various curves to the data. In the “c6” data product, there are 64 mass bins, 32 energy bins, and no resolution in the angular dimension (all angular dimensions are summed). The other data product used, “d0,” has less mass resolution, 8 bins, but the same number of energy bins and 64 angle bins (derived from separating the data from each of the 16 anodes and making 4 bins for the deflector angle; McFadden et al., 2015).

The data used in this paper covers the period 15–22 April 2018 (*L*_s_ = 159.4°–163.6°). During this time, the solar wind was quiet with only one minor Co‐rotating Interaction Region (CIR), experiencing no unusual perturbations (Lee et al., 2017). These data were chosen primarily because the orbits cut through the dusk tail at high altitude where large fluxes of heavy ions were frequently observed flowing down the tail, accompanied by significant fluxes of C^+^ ions. The data were selected as a typical test case to develop the algorithms which will be later applied to a much larger data set to look for global aspects of C^+^ loss. This date range was also chosen because manual analysis had identified the presence of C^+^ on one of the orbits, making it a test case for the development of background removal code for C^+^ extraction.

## 3 Method

The goal of this study is to resolve the vector flux of C^+^ and thereby determine the net flux leaving Mars. Calculating flux $\vec{F}$ is determined by

(1) $$\vec{F}=n\vec{v},$$

where *n* is the density and $\vec{v}$ is the velocity. These quantities can be measured using a combination of STATIC data products, the “c6” data product (32 energies × 64 mass) for density and the “d0” product (32 energies × 64 solid angles × 8 mass) for velocity. These are then multiplied to obtain flux.

**Figure 1:**
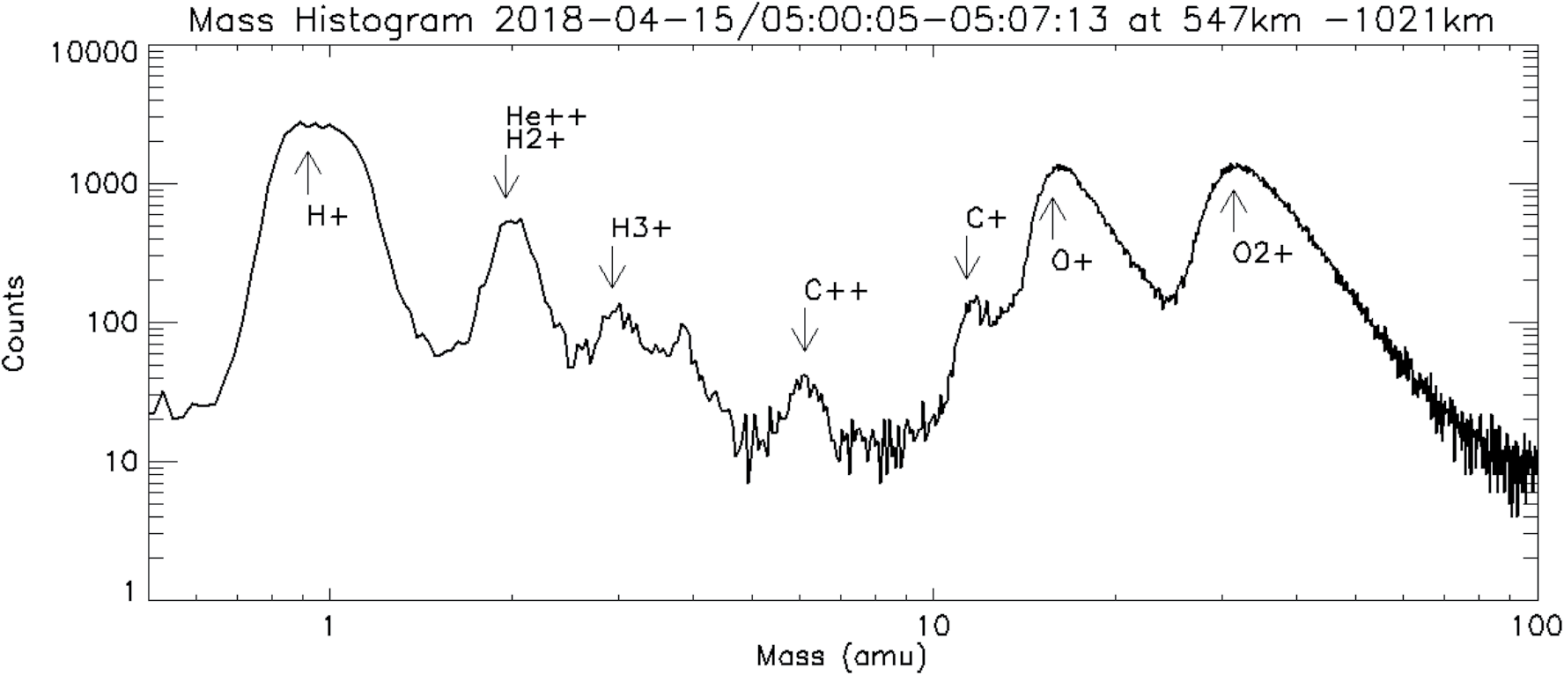
Example of mass distribution from STATIC with strong presence of heavy ions taken from 15 April 2018. The data product used is called “db” and it only has 1,024 mass bins with no resolution in any other dimension. The peaks represent ion species with distinct mass per charge values. The mass peaks are labeled in the figure.

Figure 1 shows a high‐mass resolution (1,024 mass bins, “db” data product) ion spectrum for a period of 7 min with significant heavy ion presence in the optical shadow, the shadow cast by Mars from the Sun's light. This image first shows why background subtraction and isolation must be done in order to study ion species: Each broad peak represents a distinct ion species that must be isolated from other nearby overlapping peaks to be accurately measured. The two large peaks above *M*/*Q* = 10 indicate high levels of O^+^ and O_2_^+^. While the Martian atmosphere is predominately composed of CO_2_, the ionosphere in dominated by O_2_^+^ with about 10–20% CO_2_^+^ and a few percent O^+^ on the dayside below 200 km. At altitudes greater than 250 km, O^+^ replaces CO_2_^+^ as the second most abundant ion species with O^+^ concentrations between 10% and 50% of the O_2_^+^, and CO_2_^+^ falling to a few percent of the total (Schunk & Nagy, 2000). The relative abundance of O^+^ make measuring C^+^ more difficult because C^+^ forms a small overlapping shoulder on the low‐mass edge of the O^+^ peak. Unfortunately, these high‐mass resolution distributions do not contain energy information needed to correct for detector dead time, so instead the energy‐resolved “c6” data products are used to determine density.

### 3.1 Determining Density

**Figure 2:**
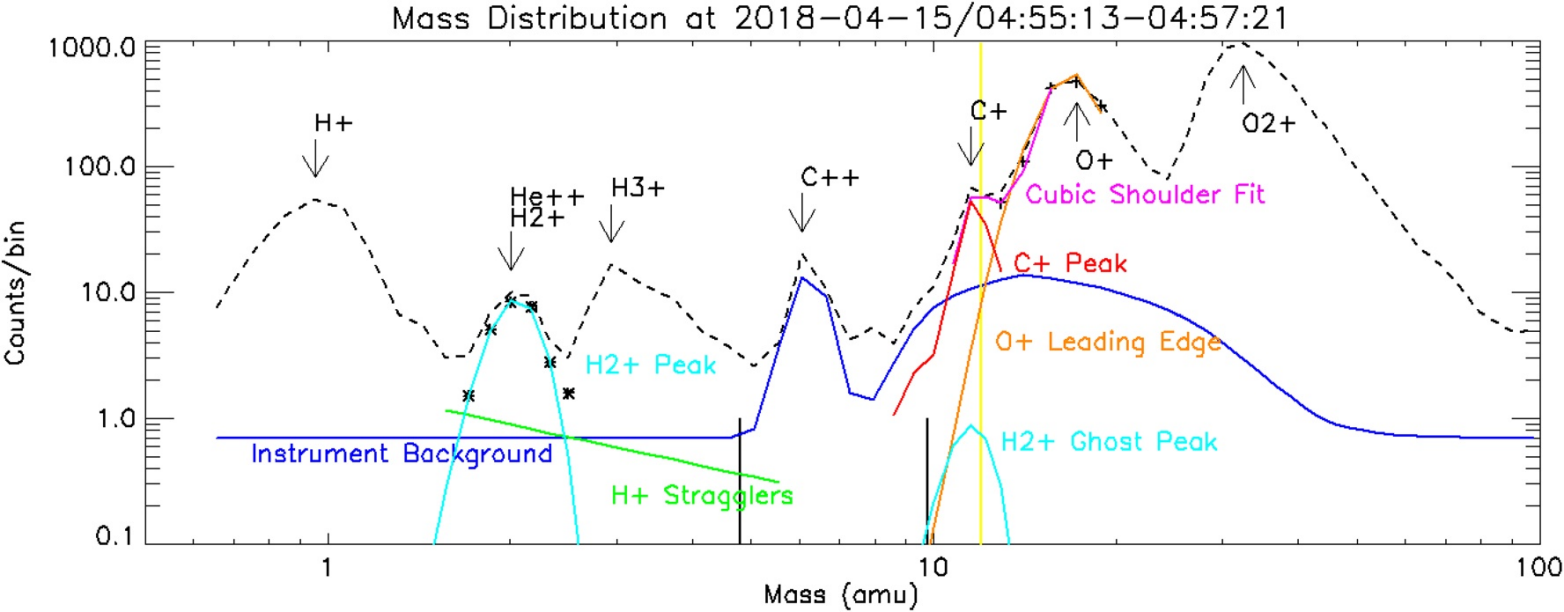
Example of background subtraction on mass distribution taken from 15 April 2018. The background subtractions shown are: Instrument background (dark blue), Gaussian fit for H_2_^+^ (cyan curve on the left), Power law fit for H^+^ stragglers (green), Gaussian fit for H_2_^+^ ghost peak (right cyan peak) Cubic fit to detect C^+^ shoulder (magenta), Gaussian fit to remove leading edge of O^+^ peak (orange). The final C^+^ peak is shown in red. The *M*/*Q* = 12 position on the *X* axis is indicated in yellow. Stars indicate the points which the left cyan peak were fit to. Pluses indicate the points which the orange peak were fit to. The black bars mark the “d0” bin used in Section 3.2. See text for more details.

In order to isolate C^+^ ions to determine their density, “c6” data are summed over energy as in Figure 2. Figure 2 subsequently shows the total counts for each TOF bin, along with various curves used in the calculation of C^+^ density, which are described in detail below. There are three sources of counts not due to carbon ions that must be removed: instrument background, a ghost peak resulting from H_2_^+^, and O^+^ overlap with the C^+^ peak handled with a fitting routine.

The instrument background (Figure 2, blue curve) is calculated separately. For these data, it consists of

1. Coincident events, where different ions create the start and stop signal in the TOF mass analyzer used to calculate mass
2. Delayed start signals from a molecular ion fragment, and
3. Internally produced sputtered C^++^ ions off the energy analyzer resulting from 15 keV secondary electrons accelerated from the start foil

Coincident events produce a nearly flat mass spectrum as seen at low mass and vary as the total ion count rate squared. Molecular fragments produce the broad shoulder below the O_2_^+^ peak, and sputtered C^++^ form a separate peak at *M*/*Q* = 6. Both of these latter backgrounds were seen in ground calibrations (McFadden et al., 2015), were modeled, and calculated using the raw data.

The proton straggling background (Figure 2, green curve) was estimated after subtraction of “instrument background” (blue curve) from the mass spectra (black dashed), then fitting a power law to two points: half the counts at the lowest point between the *M*/*Q* = 1 and *M*/*Q* = 2 peaks, and a second point between He^+^ and C^++^. This second point was chosen through manual analysis of case studies which showed that particular point was the point after the H_2_^+^ peak most frequently dominated by H^+^ straggling (though that is not the case in the data shown in Figure 2). Regardless, the procedure is not sensitive to the second point since these stragglers are only removed around the *M*/*Q* = 2 peak (causing a 10–20% correction) but is necessary since simpler estimations of this background would underestimate the H_2_^+^ peak. The resulting *M*/*Q* = 2 peak is then used to estimate the maximum H_2_^+^ ghost peak (cyan) for subtraction from the C^+^ peak.

As detailed in Figure 32 of McFadden et al. (2015), H_2_^+^ ions hitting the start carbon foil inside STATIC break into fragments, causing a delayed “stop” signal that creates a ghost peak at *M*/*Q* = 12. The ghost peak is (3.2 ± 0.3) × 10^−2^ times smaller in area than the H_2_^+^ peak and directly overlaps the C^+^ peak, requiring its removal (McFadden et al., 2015). Since He^++^ and H_2_^+^ are both *M*/*Q* = 2, an assumption must be made about the relative abundance of the two ion species before the size of the ghost peak is determined. The assumption made with these data is that the entire *M*/*Q* = 2 peak is H_2_^+^. In calculating C^+^, the conservative assumption is that the *M*/*Q* = 2 peak is all H_2_^+^; this may overestimate the background and gives a lower bound on the amount of C^+^ detected.

The background removal procedure is performed as follows, with the color of the corresponding curve in Figure 2 indicated in parentheses:

1. In order to get enough counts for the fitting routine to work, the “c6” data are summed over sliding 60‐s intervals to create a data product with 4‐s cadence and a sufficient signal‐to‐noise ratio to reliably extract C^+^
2. The instrument's background removal procedure is run to subtract off the instrument background (dark blue) from the raw data (black)
3. A cubic fit is performed on the leading edge of the O^+^ peak to determine if a shoulder is present (magenta). The method used for spotting the shoulder relies on the fitted cubic function's parameters as seen in Equation 2
(2) $$\frac{B^{2}}{AC}>3,\;f\left(x\right)=Ax^{3}+Bx^{2}+Cx+D.$$
  In order for the cubic function to have a shoulder, the derivative of the cubic polynomial (*y* = 3*Ax* ^2^ + 2*Bx* + *C*) needs to cross *y* = 0 twice, indicating it has two real roots. For a quadratic function (*y* = *ax* ^2^ + *bx* + *c*) to have two real roots, the *b* ^2^ − 4*ac* term of the quadratic formula must be greater than 0. Therefore *b* ^2^ > 4*ac* or (2*B*)^2^ > 4(3*A*)*C* or *B* ^2^/(*AC*) > 3. Its purpose is to determine which points may have C^+^ and need the full analysis and which can be skipped for lacking a C^+^ peak entirely.
4. A Gaussian is fitted to the top of the O^+^ peak (the points used in this fit are indicated with a plus in Figure 2) and extrapolated down the leading edge of the peak past the C^+^ ion peak (orange)
5. A power law fit is made over the *M*/*Q* = 2 peak to account for the tail of the H^+^ peak affecting the measurement of H_2_^+^/He^++^ (green)
6. A Gaussian fit is performed on the *M*/*Q* = 2 peak (the points used in this fit are indicated with a star in Figure 2) after background from the previous step has been removed from the spectra (left cyan) assuming that the entire *M*/*Q* = 2 peak is H_2_^+^
7. The coefficients of the previous fit are used to determine the size of the H_2_^+^ ghost peak by scaling the curve's integrated area by a factor of 30 and shifting the peak over to *M*/*Q* = 12 (right cyan; McFadden et al., 2015)
8. The total count of C^+^ ions is compared to 2.2 times the Poisson error of the original *M*/*Q* = 12 peak count with background. Data points with counts beneath this threshold will be removed from the data set to account for uncertainty in the data. This value was empirically selected to remove false positives in regions where detailed manual analysis of the data indicated statistical fluctuations were producing random false positives

The result of this process is indicated in red and, in the measurement shown in Figure 2, shows that the shoulder is predominantly from C^+^ ions.

The density calculation for “c6” data depends on energy of the ions, but the calculated background curves are done with all of the data is summed over the energy dimension. Performing the fitting routine on each individual bin of energy can fail if the number of counts is too small. Therefore, in order to apply the background removal across each energy bin, the fit parameters from the summed distribution are scaled to the height of the peak they were determined from in each energy bin (H^+^ for the power law fit, H_2_^+^ for the ghost peak fit, O^+^ for the leading‐edge fit). The instrument background has already been calculated as a function of energy. The resulting data product can finally be integrated to determine C^+^ ion's true density.

### 3.2 Determining Velocity

Given that the “c6” data product has no angular resolution, the data product “d0” must be used to calculate the plasma's velocity. “d0” has less mass bins than the “c6” data, making the same fitting‐based background removal procedure done on “c6” data impossible. Fortunately, one of the mass bins in “d0” only measures the low‐mass leading edge of the C^+^ peak, i.e., that portion of the C^+^ peak that is not impacted by the leading edge of O^+^ (the mass range of this is indicated in Figure 2 with two short black bars). The velocity determined from the “d0” product for this mass bin is a good proxy for C^+^ velocity as “d0” products have the same background removal as shown in the dark blue curve in Figure 2, although “d0” does not include ghost peak removal. The velocity obtained by integrating this background‐subtracted, energy‐resolved resultant product just needs to be corrected for spacecraft velocity before being combined with density to produce the C^+^ ion flux.

## 4 Results and Analysis

### 4.1 Results

**Figure 3:**
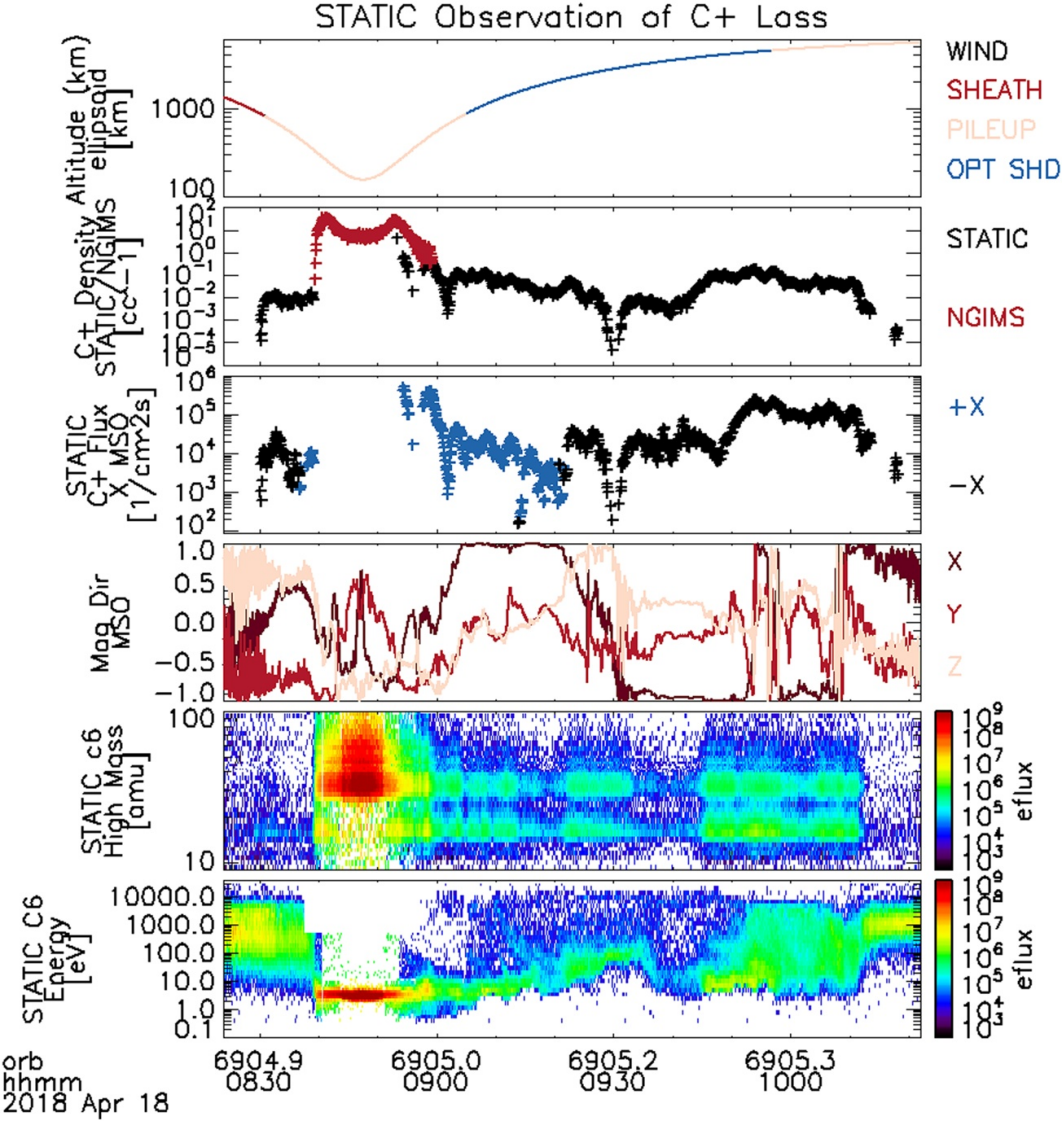
Subsection of results of C^+^ ion flux from 18 April 2018. The periapsis of this orbit occurs at around (45°S, 300°E). From top to bottom, the graphs show: MAVEN's altitude as a function of time, the color coding showing where in the structure of the ionosphere the craft is; C^+^ ion density over time, the red being data from NGIMS and the black being results from STATIC; C^+^ flux, the black being tailward and the blue being sunward; Mars' magnetic field direction as a function of time in the MSO (Mars‐Sun‐Orbit) frame; and the STATIC “c6” data product mass and energy readout. The statistical error on these results is too small to show graphically.

These results are from processing 40 orbits from 15 April 2018 to 22 April 2018 using the above procedure. Figure 3 shows a subset of the results from 18 April 2018 that is typical of the orbits analyzed. The first panel shows the altitude of the spacecraft with color indicating the nominal range covered by different regions of the Martian magnetosphere. The second panel shows the measured density of C^+^ ions from STATIC using the process described above compared to the NGIMS level 2 abundance. NGIMS is generally capable of measuring cold C^+^ ions below 500 km, although sensitivity can vary above 200 km depending on temperatures and winds (Mahaffy et al., 2015). Above 500 km, NGIMS’ narrow FOV combined with off‐pointing from the combined ion flows and ram flows, generally preclude C^+^ observations. Because STATIC attenuators and modes change frequently at periapsis, causing changes in the angular resolution, energy resolution, and geometric factor, the program used to create the averaged data product is not always applicable. This means C^+^ fluxes are usually only obtainable above altitudes of 300 km. Even without these operational issues, background from molecular dissociation of O_2_^+^ is generally too high to detect C^+^ below 300 km using STATIC. The existence of this narrow region of overlap over 300–500 km between the NGIMS and STATIC measurements provides support for the validity of our background removal procedure. Given the different field of view of the two instruments, the results are not expected to be identical but consistency within known errors provides confidence in the procedure's accuracy. The third panel shows the C^+^ ion flux in the *X* direction in the MSO (Mars‐Sun‐Orbit) frame; the negative *X* direction in this frame is generally the direction of the magnetotail. The fourth panel shows the magnetic field direction in the MSO frame. The fifth and sixth panels show the raw “c6” STATIC mass and energy data, respectively.

### 4.2 Analysis of the Behavior of C^+^

This limited study measures the average C^+^ ion outflow rates down the tail above 3,000‐km altitude at a one sigma range of 9 × 10^3^ to 1 × 10^5^/cm^2^ s. To our knowledge, no other estimates of C^+^ escape rates have been published at Mars, but we can compare to published estimates of neutral C escape. Fox and Bakalian (2001) calculated the global photochemical C escape rate just above the exobase region at Mars (200‐km altitude) to be 1 × 10^5^/cm^2^ s. The outflow rates of C^+^ measured by STATIC in our study are comparable, although a side‐by‐side comparison must be made with caution. The escape rates from Fox and Bakalian (2001) are calculated above the exobase region, while those from STATIC are made at altitudes >3,000 km in the tail only. The total loss rates of neutral versus charged carbon may thus be very different when one takes into account factors such as the surface area that carbon can be lost through, and whether carbon is lost in specific regions of the planetary environment. As an example, photochemical loss of C occurs mostly on the dayside (Fox & Bakalian, 2001), while loss of C^+^ ions occurs mostly in the magnetotail region in our study. Nonetheless, our results suggest that the loss of C^+^ ions may be important in the total carbon escape budget of Mars, and a more detailed study is justified for future work.

Although for this study the downtail fluxes were primarily observed in eclipse, the STATIC team has observed escaping C^+^ flows extending into the sunlit outer layer of the Martian magnetotail. The detectable C^+^ signatures disappear, as do the O_2_^+^ signals, as the spacecraft enters the downstream tail magnetosheath. In contrast, O^+^ ions often continue into the magnetosheath, formed upstream of MAVEN by photoionization and charge exchange of the neutral oxygen corona (Rahmati et al., 2015). Lastly, for completeness, we mention that a separate distinct dayside pickup plume, with several heavy ion species and energies up to 10 of keV, may also be detected in the sheath near the terminator at high altitudes; this signature does not overlap with the C^+^ tailward flowing ions reported here.

A detectable signal of C^+^ is observed about 27% of the time for studied orbits, always concurrent with measurement of O^+^ and O_2_^+^. Detection occurs when O^+^ and O_2_^+^ densities are high. The C^+^ densities scale roughly with the O^+^ outflows, forming a small shoulder on O^+^ peak (Figure 1) with an average density approximately 1/70 the size of O^+^. The lower rate of detected C^+^ flows, in comparison to O^+^, appears to be just a sensitivity issue, where C^+^ counts drop below our statistically determined threshold for a valid event, as specified in Section 3.1.

In this study, the tailward C^+^ flows are primarily observed at >3,000 km, although they occasionally are seen down to 1,000 km. At altitudes <2,000 km, the flows generally have a sunward velocity component (see Figure 3, third panel). A detailed study of the full velocity, and its relation to the magnetic field and altitude is left to a future study with a larger data set. The observed altitude dependence is likely the result of the limited orbit geometry for this study. The high‐altitude measurements are either on open or draped flux tubes allowing ions to escape down the tail. Acceleration to escape velocities is either from solar wind convection electric fields (up to keV energies), or from a combination of ambipolar fields and ion heating at the ionosphere‐sheath shear‐flow interface near the terminator. The latter produces a cold ion outflow along the magnetic field characteristic of most events in this study. Higher‐energy outflows of pickup ions are also observed but not well represented in this limited 1‐week data set.

There are trends in this limited data set that indicate the cold C^+^ outflows increase as MAVEN approaches the edge of the optical shadow. An example of this appears in Figure 3, panel 3 around 09:50. In contrast, the density, on average, appears to be more nearly constant. Since these ions are beam‐like, the average velocity of C^+^ ions down the tail must increase as MAVEN approaches the tail‐sheath boundary. In addition, the energy of C^+^ is roughly the same energy as other heavier ions, O^+^ and O_2_^+^. Therefore, all heavy ions are traveling at different velocities and we rule out a time‐of‐flight selection effect. Instead, this suggests greater acceleration at higher altitudes at the terminator where ions are injected down the tail. Since these are cold ion beams, we expect either an altitude dependence of tailward ambipolar electric fields, or greater heating at the flow shear boundary, with the latter likely due to wave turbulence produced by the ionosphere‐sheath shear flow. A future study will expand the method of calculating C^+^ fluxes represented here to the rest of the STATIC data set in order to confirm that C^+^ outflows are most intense near the sheath‐tail boundary.

The C^+^ ions with a sunward velocity component at lower altitudes (<2,000 km, Figure 3, panel 3) appear to be the result of orbit geometry and closed flux tube relaxation. The lower altitude observations are in the southern hemisphere and post‐dusk sector. The southern hemisphere has the strongest crustal fields which are known to reconnect to the solar wind interplanetary magnetic field (IMF) on the dayside producing open flux tubes whose footpoints eventually rotate to the nightside (Harada et al., 2018). These open flux tubes will reconnect in the tail, forming extended closed crustal flux loops, similar to Earth's closed flux loops in the plasma sheet. These flux loops will relax by dipolarization and interchange instability as observed in the Earth's magnetotail (Birn et al., 2004) producing a net Mars‐ward flow which will have a positive sunward component as observed.

**Figure 4:**
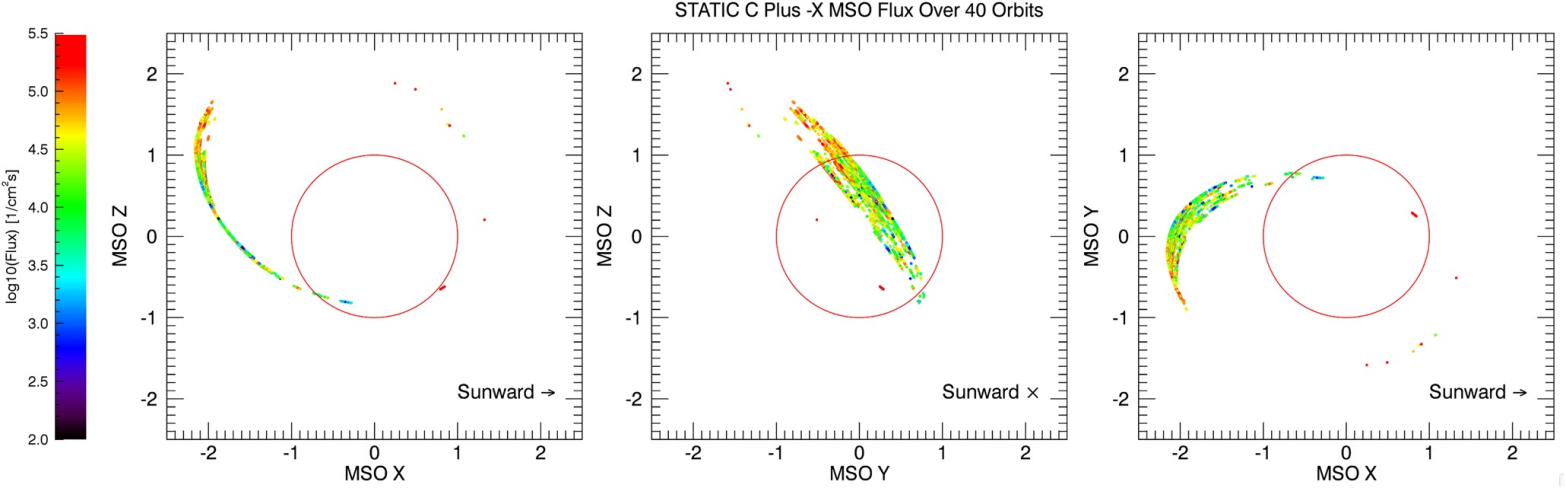
C^+^ ion flux measurements in the negative *X* direction in the MSO (Mars‐Sun‐Orbit) frame plotted by location (the positive *X* events have been removed due to the log scale used). Note the high density of measurements in the optical shadow of the planet as well as near the terminator.

While this data set study has been used to test the C^+^ extraction procedures, the results indicate there is a significant, consistent presence of C^+^ that necessitates a larger study of C^+^ ions in the Mars system. Both sunward and tailward flows are observed, along with trends in flux direction and magnitude that suggest the configuration of spatial variations in C^+^ energization. Figure 4 shows the C^+^ flux in the negative *X* direction in the MSO frame. The figure shows larger flows outside shadow as MAVEN approaches the tail‐sheath boundary as mentioned above. The cluster of points on the dayside corresponds to pickup C^+^ ions, which the procedure can occasionally detect. The figure also shows that only a small portion of the Mars system has been sampled in this study, mostly in the optical shadow. Therefore, a more elaborate study of several years of STATIC data is needed to build a more comprehensive picture of carbon ion losses.

## 5 Conclusion

Understanding the history on Mars requires an understanding of the history of carbon on Mars. To understand carbon on Mars is to understand its escape from the atmosphere. Various instruments aboard MAVEN are capable of measuring ions at Mars, but only STATIC provides the sensitivity and capabilities to measure C^+^ ion fluxes. This study serves to test a method of measuring C^+^ ions using MAVEN's STATIC instrument and justify a larger study using the method.

A background removal procedure was used on 40 orbits of data from 15 April 2018 to 22 April 2018 to produce the result seen in Figures 3 and 4, showing a consistent C^+^ ion flux loss down the tail on the order of 10^5^/cm^2^ s^1^ in all 40 orbits, making it comparable to results from neutral carbon escape found by Fox and Bakalian (2001). This limited study demonstrates that tailward C^+^ fluxes were frequently observed inside the optical shadow above 3,000‐km altitude. At lower altitudes, below 500 km, derived C^+^ densities can be compared to those from the NGIMS instrument, and the observed consistency within known errors provides confidence in the STATIC results. Broader trends, like C^+^ fluxes scaling with O^+^ fluxes and sunward directed C^+^ fluxes below 2,000 km have been noted but not investigated further in this focused study.

The procedure outlined here to calculate C^+^ fluxes from the STATIC instrument produces accurate, consistent results while also showing a significant measurement of C^+^ ion flux in the optical shadow that necessitates a more comprehensive study over the full MAVEN mission. These new measurements of C^+^ fluxes at Mars will contribute more understanding to the picture of carbon escape on Mars.

## Data Availability

All data from STATIC, the MAVEN magnetometer, and NGIMS are available through NASA's Planetary Data System at https://pds-ppi.igpp.ucla.edu/search/?t=Mars&sc=MAVEN&facet=SPACECRAFT_NAME&depth=1. All data were version V02 and revision R04‐R06, depending on orbit.
